# A Sparse Perspective for Direction-of-Arrival Estimation Under Strong Near-Field Interference Environment

**DOI:** 10.3390/s20010163

**Published:** 2019-12-26

**Authors:** Longhao Qiu, Tian Lan, Yilin Wang

**Affiliations:** 1 Acoustic Science and Technology Laboratory, Harbin Engineering University, Harbin 150001, China; qiulonghao@hrbeu.edu.cn (L.Q.); wangyilin@hrbeu.edu.cn (Y.W.); 2 Key Laboratory of Marine Information Acquisition and Security (Harbin Engineering University), Ministry of Industry and Information Technology, Harbin 150001, China; 3 College of Underwater Acoustic Engineering, Harbin Engineering University, Harbin 150001, China; 4 Qingdao Haina Underwater Information Technology Co., Ltd., Qingdao 266500, China

**Keywords:** array signal processing, DOA estimation, sparse bayesian learning, near-field interference

## Abstract

Direction of arrival (DOA) estimation via sensor array is a crucial component of any passive sonar signal processing technology. In certain practical applications, however, the interested far-field targets are frequently affected by near-field interference, which may result in degradation of DOA estimation. Aiming at the direction estimation problems of far-field targets under strong near-field interference, a unified sparse representation model of far-field and near-field hybrid sources is constructed according to the various correlations in steering vectors between the planar wave and spherical wave in this paper. A high-resolution spatial spectrum reconstruction algorithm based on a sparse Bayesian framework is then exploited to constrain the energy of near-field interference in the preset near-field steering vector over-complete dictionary, thus ensuring the accurate detection and estimation of far-field targets. An expectation-maximization (EM) algorithm approach is introduced to estimate the number of sources and noise power iteratively, which will reduce the dependence of the algorithm on such prior information. Several state-of-art algorithms are mentioned and discussed (Minimum Variance Distortionless Response (MVDR) method, Multiple Signal Classification (MUSIC) algorithm and conventional beamforming (CBF) algorithm) to compare with the one proposed in this manuscript that achieves higher accuracy of estimation and resolution under low SNR level with limited samples, which is verified by simulation and for the results obtained in an experimental case study.

## 1. Introduction

Direction of arrival (DOA) estimation via an acoustic sensor array mounted on underwater vehicles is an important research subject in the field of passive sonar signal processing [1,2]. It is well known that, in order to achieve high performance of DOA estimation, a large aperture array is required. Various high-resolution algorithms, such as the Minimum Variance Distortionless Response (MVDR) [3], the Multiple Signal Classification (MUSIC) [4], the Sparse Spectrum Fitting (SpSF) [5] are currently adopted methods for the ability to break through the Rayleigh Resolution Limit determined by the array aperture. However, due to the complex practical underwater acoustic environment, the received signals of sonar arrays could easily be affected by noises and interferences. The radiated noise levels of interested targets are low due to long-distance propagation, resulting in very low signal-to-noise (SNR) ratio. The local platform in motion is also a very complex noise source encompassing propeller noise, vibration noise, and other interfering elements [6]. Near-field strong interferences from tow-vessel noise and the platform’s self-noise affect the accuracy of DOA estimation and may even completely obscure certain weak targets. There is a demand for innovative array signal processing methods to achieve high-resolution in the presence of local near-field strong interferences.

Extant interference suppression methods usually rely on the adaptive noise canceller (ANC) [7,8,9] or spatial filter [10,11]. Adaptive noise canceller is widely exploited to suppress the interference components from interested signals. The basic idea of ANC is to make use of a “primary” input containing the corrupted signal and a “reference” input containing noise or interference correlated in some way. The reference input is adaptively filtered and subtracted from the primary input to extract the pure signal. Since ANC was introduced by Bernard Widrow [12], many linear and nonlinear filters are developed to estimate the filter parameters and can provide satisfactory performance under general conditions [13,14]. To apply these algorithms to near-field interference suppression, the accurate measurement or estimation of interference is required as the filters’ input, which can be very difficult to secure in practice. In order to apply the ANC algorithms to near-field interference suppression, serval focused beamformers have been developed to locate and extract near-field sources [15,16,17], but some important parameters such as the side-lobe level, array gain, and width of the main lobe are difficult to compromise. Interference source characteristics, such as position and intensity can be time-varying, which leads to impure reference signal extraction, further limiting the practical application of the adaptive interference cancellation algorithm.

The matrix filter is a kind of spatial filter that passes sector-of-interest signals and suppresses out-of-sector interference. Researchers have developed numerous matrix filters with nulls in the stopband to suppress interference in the far-field. [18,19,20,21,22]. Vaccaro [18] introduced a set of criteria for designing a matrix filter. MacInnes [20] design a matrix filter in an efficient manner by formulating the design procedure as a rank-deficient linear-squares problem. Yan [22] further reformulated the matrix filter design as a second-order cone (SOC) programming. The uncontrollable transitional band is an inevitable problem and the abilities of the aforementioned matrix filters in interference suppression are limited by stopband attenuation. When it comes to near-filed interference, the correlation of the array manifold between the spherical wave and plane wave is highly complicated, which makes it difficult to compromise the passband and stopband of the matrix filter. The matrix filter with nulling (MFN), which is driven by the received data and is capable of forming nulls adaptively, can also suppress strong interference [11,23]. However, a blind region persists in the far-field as near-field interference is suppressed. Other spatial filters have also been proposed for interference suppression [24,25,26] while cannot achieve satisfactory performance when the signal of interest and the interference have a similar orientation.

Sparse signal recovery (SSR) based methods have been applied to solve the problem of DOA estimation of far-field signals in recent years [27,28,29,30,31]. It has been validated that via exploiting spatial sparsity, these SSR-based methods exhibit remarkable superiority in resolution and robustness to noise and snapshot number. L1 norm based singular value decomposition (L1-SVD) [32] took L1 norm as the penalty function for sparse constraints and a convex optimization problem is solved to estimate the DOA of far-field incoming signals. A reweighted L1-norm minimization subject to an error-constrained L2 norm is designed to determine the DOA estimates under the coexistence of mutual coupling and nonuniform noise [33]. A joint nonnegative SBL procedure was developed to estimate direction-of-arrival (DOA) for wideband signals in [34]. The off-grid error and non-uniform noise problem are discussed in [35] by iteratively estimating the signal power and the covariance matrix of unknown non-uniform noise. However, the ability of SSR based DOA methods among strong near-field interferences has not been proven yet.

The direction estimation problems of far-field targets under near-field strong interference were investigated in this study by analyzing the differences in steering vectors between far-field and near-field signals. The near-field interference is modeled in this paper as a spherical wave rather than a Fresnel Approximation. A unified sparse representation model of far-field and near-field hybrid sources is constructed considering the correlation difference between the planar waveguide vector and the spherical waveguide vector. A sparse Bayesian learning algorithm to separate far-field and near-field signals is then proposed and the expectation-maximization (EM) algorithm is exploited to provide the DOA estimation automatically without preset regularization parameters and the prior knowledge of the number of sources. The proposed method enhances the ability of sparse signal processing for weak far-field target localization in the strong near-field interference environment.

The remainder of this paper is organized as follows. Section 2 introduces the signal model and correlation analysis of steering vectors between near and far-field. Section 3 gives an overview of related works. In Section 4, the separation method of far-field and near-field sources is proposed and then the DOA estimator based on the sparse Bayesian learning framework is presented. Section 5 carries out the simulation analysis and experimental results, which validate the interference separation and bearing estimation accuracy of the proposed method. Finally, conclusions are summarized in Section 6.

## 2. Problem Description

### 2.1. Signal Model

Suppose there are K narrowband signals (including *K*_1_ far-field targets and *K*_2_ near-field interferences) impinging on an array composed of *M* sensors. The sensor spacing of the uniform linear array (ULA) is d and the aperture of the array is expressed as *D* = (*M* − 1) *d*. For the *k*th (*k* = 1, …, *K*_1_) signal in the far-field, the wavefront of acoustic propagation is assumed to be a plane. In this case, the amplitudes of the signals are all the same on each sensor; the phase differences between the sensors are determined by the angle of targets. For the other kth (*k* = *K*_1_ + 1, …, *K*) interferences in the near-field, the acoustic wavefront is assumed to propagate on the spherical wavelet. The locations of the interferences relative to the reference sensor are determined by two-dimensional (2D) parameters which are described in polar coordinates as $\left(r_{k},\theta_{k}\right)$ and in Cartesian coordinates as $\left(x_{k},y_{k}\right)$, where $x_{k}=r_{k}\cos\theta_{k},\;y_{k}=r_{k}\sin\theta_{k}$. 

The receiving interference amplitudes at different sensors are different. The phase difference between sensors is determined by the angle and distance of the impinging signals. Therefore, with the presence of near-field interference, the received signal of the mth (*m* = 1, …, *M*) sensor can be expressed as follows:

(1) $$p_{m}(t)=\sum_{k=1}^{K_{1}}s_{k}(t-\tau_{mk})+\sum_{k=K_{1}+1}^{K}\frac{r_{k}}{r_{mk}}s_{k}(t-\tau_{mk})+n_{mp}(t),\;m=1,2,\ldots ,M,$$

where $n_{mp}(t)$ is additive noise and $\tau_{mk}$ is the time delay associated with the kth source between the mth sensor and the reference point. $r_{mk}$ represents the distance between the kth source and the mth sensor, which satisfies $r_{mk}^{2}=r_{k}^{2}+{\left(m-1\right)}^{2}d^{2}-2r_{k}(m-1)d\cos\theta_{k}$. Let $s_{F}(t)={\left[s_{1}(t),\ldots ,s_{K_{1}}(t)\right]}^{T}$ and $s_{N}(t)={\left[s_{K_{1}+1}(t),\ldots ,s_{K}(t)\right]}^{T}$ denote far-field signals and near-field interferences, respectively. Equation (1) can then be rewritten in matrix form as:

(2) $$X(t)=\left[\begin{array}{cc} A_{F}(\theta ) & A_{N}(r,\theta ) \end{array}\right]\left[\begin{array}{c} s_{F}(t)\\ s_{N}(t) \end{array}\right]+N(t),$$

where $A_{F}(\theta )=[a_{F}(\theta_{1}),\ldots ,a_{F}(\theta_{K_{1}})]$ represents the array manifold of the far-field source signal and $a_{F}(\theta_{k})$ is the steering vector for the plane wave; $A_{N}(r,\theta )=[a_{N}(r_{K_{1}+1},\theta_{K_{1}+1}),\ldots ,a_{N}(r_{K},\theta_{K})]$ represents the array response of near-field interference where $a_{N}(r_{K},\theta_{K})$ is the steering vector for the spherical wave and N(t) is additive white noise. For a uniform linear array (ULA), the steering vector can be written as:

(3) $$\begin{array}{l} a_{F}(\theta_{k})={\left[1,\ldots ,e^{-j\frac{2\pi d\cos\theta_{k}}{\lambda }},\ldots ,e^{-j\frac{2\pi (M-1)d\cos\theta_{K_{1}}}{\lambda }}\right]}^{T},k\;=1,\ldots ,K_{1},\\ a_{N}(r_{k},\theta_{k})={\left[1,\frac{r_{k}}{r_{2k}}e^{-j\frac{2\pi (r_{2k}-r_{k})}{\lambda }},\ldots ,\frac{r_{k}}{r_{Mk}}e^{-j\frac{2\pi (r_{Mk}-r_{k})}{\lambda }}\right]}^{T},k\;=K_{1}+1,\ldots ,K. \end{array}$$

### 2.2. Fresnel Approximation Model of Near-field

$r\gg D^{2}/\lambda$ is usually taken as the far-field condition in array signal processing, but the boundary between the far-field and near-field is not clearly defined. In the scenarios where both far-field targets and near-field interferences coexist, generally, the near-field source model is simplified. It is typically assumed that when the near-field source is located in the Fresnel region ($r\in [0.62{\left(D^{3}/\lambda \right)}^{1/2},2D^{2}/\lambda ]$) of the receiving array, the complex envelope of the received signal from different sensors is approximately equal, i.e., $r_{k}/r_{mk}\approx 1$. In addition, the phase difference between different sensors can be approximated by the second-order Taylor expansion:

(4) $$-\frac{2\pi \left(r_{mk}-r_{k}\right)}{\lambda }\approx -2\pi \frac{\left(m-1\right)d}{\lambda }\sin\theta_{k}+\pi \frac{{\left(m-1\right)}^{2}d^{2}}{\lambda r_{k}}\cos^{2}\theta_{k}.$$

Under this assumption, the steering vector $a_{N}(r_{k},\theta_{k})$ can be simplified as follows:

(5) $$\begin{array}{l} a_{N}\left(r_{k},\theta_{k}\right)\approx {\left[1,\ldots ,e^{j\left(m\alpha_{k}+m^{2}\beta_{k}\right)},\ldots ,e^{j\left(\left(M-1\right)\alpha_{k}+{\left(M-1\right)}^{2}\beta_{k}\right)}\right]}^{T},\\ \alpha_{k}=-2\pi \frac{d}{\lambda }\sin\theta_{k},\beta_{k}=\pi \frac{d^{2}}{\lambda r_{k}}\cos^{2}\theta_{k}. \end{array}$$

Equation (5) is a so-called Fresnel approximation and its significance lies in pointing out that the wavefront of the spherical wave received by the array is no longer a plane and can be approximated to quadric surface form in the Fresnel region. The phase difference between different sensors can be separated into two parts via Fresnel approximation, the first term of which is independent of the distance of sources. This property provides a great deal of convenience to relative array signal processing. However, model errors are inevitably introduced by omitting the higher-order Taylor expansion terms and neglecting the amplitude difference among sensors. Errors in this case are related to the specific location of near-field interference.

**Figure 1:**
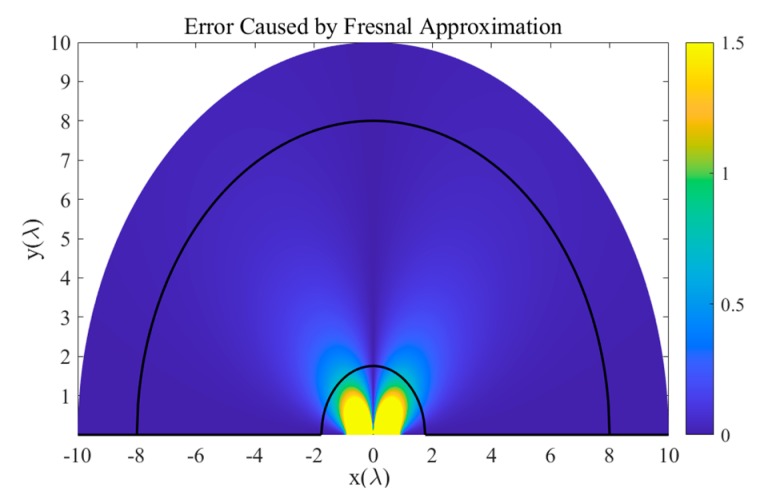
Phase difference error introduced by Fresnel approximation.

As shown in Figure 1, the error simulation of the phase difference between two elements caused by Fresnel approximation was carried out in this study in a 9-element symmetric uniform linear array. This reflects the difference between the two sides of Equation (4).

It is assumed that the reference sensor is at the coordinate origin and the array is placed along the *x*-axis. The two black solid lines in the figure represent the Fresnel area of the receiving array. The *x* and *y*-axis directions are the end-fire and normal direction of the ULA, respectively. Along the *x*-axis direction, the phase difference among different sensors at any distance only depends on the sensor spacing and there is no approximate error. In other areas of the figure, model error due to Fresnel approximation gradually emerges and decreases as distance increases. In the normal direction of the array, the phase difference error between the symmetrical sensors of the origin is zero; there are still errors between the asymmetrical sensors introduced by the approximation. 

The Fresnel zone determined by the empirical formula does not take the model errors caused by the impinging signals at different locations to the array into account. In other words, the Fresnel regional boundary is not an appropriate basis for distinguishing the near-field from the far-field. To minimize the influence of model errors, the approximate Equation (3) can be used to model the near-field interferences.

### 2.3. Correlation Analysis of Steering Vectors Between Near and Far-field

The similarity between plane and spherical wave is closely related to the specific position of the sound source relative to the receiving array. Liang [24] analyzed the correlation of steering vectors between the spherical wave and plane wave from the perspective of adaptive beamforming, then derived an approximate analytical expression of the correlation coefficient based on the stationary phase principle. The correlation coefficient was shown to be related to the array aperture, the source location, and the signal wavelength. The near-field source located at a certain position in space has a strong correlation coefficient with the far-field source located at a certain width of the sector area behind it. The energy of the near-field point source presents a certain “space broadening” when viewed from the far-field perspective. Near-field interference has greater sector influence than far-field interference and is more difficult to suppress. In this paper, an intuitive physical quantity describing the correlation between steering vectors of plane wave and the spherical wave is defined as follows:

(6) $$\rho \left(r,\theta \right)=\underset{\theta_{F}}{\max}\left|\frac{a_{N}^{H}\left(r,\theta \right)a_{F}\left(\theta_{F}\right)}{\left|a_{N}\left(r,\theta \right)\right|\left|a_{F}\left(\theta_{F}\right)\right|}\right|$$

where ρ(r,θ) is the maximum correlation coefficient between the near-field source located at (r,θ) and the far-field full-space steering vector. The closer ρ(r,θ) is to 1, the more closely the steering vector of the spherical wave of the near-field source resembles the far-field planar wave in a certain direction. Under the condition ρ(r,θ)→1, the near-field source can be regarded as a far-field source. Specifically, the source located in the end-fire direction of the array has a completely correlated steering vector unrelated to the distance parameter. In fact, the far-field steering vectors constitute an over-complete dictionary. Any near-field steering vector can be accurately expressed by a linear combination of the far-field steering vectors:

(7) $$a_{N}\left(r,\theta \right)=\int_{\theta_{b}}^{\theta_{s}}\xi \left(\theta \right)a_{F}\left(\theta \right)d\theta ,$$

where $\theta_{b}$ and $\theta_{s}$ denote the lower and upper integration bounds. Equation (7) indicates that a closer ρ(r,θ) to 1 makes for a smaller the difference $\left|\theta_{s}-\theta_{b}\right|$ between the upper and lower bounds of the integral. $\left|\theta_{s}-\theta_{b}\right|$ increases as ρ(r,θ) approaches 0, which indicates that more far-field steering vectors need to participate in representing the near-field interference steering vectors. 

## 3. Related Work

The conventional beamforming (CBF) is that many sensors coherently combine the received signal, thus producing a sufficient array gain for target detection (whether a target is present or not). To achieve spatial filtering, the received signal is multiplied by the steering vector $a_{F}\left(\theta \right)$ based on the phase delay of observation, the complex output of CBF is then written as:

(8) $$Y\left(\theta ,t\right)=a_{F}^{H}\left(\theta \right)X\left(t\right),$$

where *H* denotes the conjugate transpose. The spatial power as a function of the steering angle is given by:

(9) $$P_{CBF}\left(\theta \right)=E\left[{\left|Y\left(\theta ,t\right)\right|}^{2}\right]=a_{F}^{H}\left(\theta \right)Ra_{F}\left(\theta \right),$$

where $R=E\left[X\left(t\right)X^{H}\left(t\right)\right]$ is the covariance matrix of receiving data and E[•] represents the expectation. In practical applications, R is estimated by the average over limited samples:

(10) $$\hat{R}=X\left(t\right)X^{H}\left(t\right)/T,$$

where *T* denotes the number of snapshots. CBF is robustness against mismatch between the assumed and the actual signal steering vector (such as the uncertainty of sensor position and the small size of samples). As is well known, CBF suffers from poor resolution because of the wide beam. The high sidelobe also makes it difficult to detect a weak signal in the presence of strong interferences.

MVDR beamformer minimizes the beam output power in the noise and interference direction while keeping a distortionless response in the observed direction. The beamforming weight of MVDR is optimized by:

(11) $$\begin{array}{l} \underset{\omega }{\min}\omega^{H}R\omega \\ s.t.\;\omega^{H}a_{F}\left(\theta \right)=1. \end{array}$$

Using the Lagrangian multiplier, the minimization yields the following ω and the spatial power estimation:

(12) $$\begin{array}{l} \omega_{MVDR}=\frac{R^{-1}a_{F}\left(\theta \right)}{a_{F}^{H}\left(\theta \right)R^{-1}a_{F}\left(\theta \right)},\\ P_{MVDR}\left(\theta \right)=\omega_{MVDR}^{H}R\omega_{MVDR}=\frac{1}{a_{F}^{H}\left(\theta \right)R^{-1}a_{F}\left(\theta \right)}. \end{array}$$

MVDR beamformer produces a null response in the direction of interferences and hence can provide a far better resolution and stronger interference suppression capability than CBF. In practice, there is always an error between the estimated covariance matrix in Equation (10) and the true covariance matrix because of the limited snapshots. When the number of snapshots is insufficient, the estimated covariance matrix can often be singular and cause an ill-conditioned problem of a matrix inverse. MVDR is also known to be sensitive to the signal mismatch problem.

MUSIC is an eigenvector-based algorithm. For a stationary isotropic noise environment, where a sufficient number of snapshots can be used to estimate covariance matrix accurately, the *M* eigenvalues and the corresponding eigenvectors can be obtained by:

(13) $$R=\sum_{k=1}^{K}\lambda_{k}u_{k}u_{k}^{H}+\sigma_{n}^{2}\sum_{k=K+1}^{M}u_{k}u_{k}^{H}=\sum_{k=1}^{K}\lambda_{k}u_{k}u_{k}^{H}+\sigma_{n}^{2}I.$$

The *K* largest eigenvalues and the corresponding eigenvectors can be associated with uncorrelated sources (including targets and interferences). $U_{S}=[u_{1},\ldots ,u_{k}]$ and $U_{N}=[u_{K+1},\ldots ,u_{M}]$ denotes the signal subspace and noise subspace, respectively. The spatial spectrum of MUSIC can then be written as:

(14) $$P_{MUSIC}\left(\theta \right)=\frac{1}{a_{F}^{H}\left(\theta \right)U_{N}U_{N}^{H}a_{F}\left(\theta \right)}$$

MUSIC makes full use of the orthogonal characteristics of the signal subspace and the noise subspace and hence achieves super-resolution and allows for better performance of interference rejection. Similar to the MVDR algorithm, MUSIC also suffers from insufficient snapshots. The source number is also assumed to be prior information to separate the signal subspace from the noise subspace reliably. 

## 4. DOA Estimation Under Near-Field Interference Based on Sparse Bayesian Learning

### 4.1. Influence of Near-Field Interference on Sparse Reconstruction Algorithms

Previous researches have proven the sparse reconstruction algorithm as applied to far-field DOA estimation to be rational. Numerous high-resolution DOA estimation algorithms based on sparse signal reconstruction have been proposed to date including the L1-singular value decomposition (L1-SVD) and Spare Bayesian Learning (SBL). Sparse signal reconstruction algorithms can manage coherent signals and perform well even when snapshots are scarce and SNR is low.

The sparse representation model assumes that the array received signal consists of linear combinations of a few far-field steering vectors in A(Θ) and the isotropic noise, where A(Θ) consists of sufficient far-field steering vectors so that the quantization error can be neglected. As the basis of A(Θ) in the dictionary is designed for the far-field, when near-field interferences exist, A(Θ) is no longer over-complete and not enough to describe the array response to the near-field source. In this case, more irrelevant vectors (steering vectors of unreal signal direction) in A(Θ) become involved in the fitting of the response vector of the near field source. The sparse model mismatches and the influence of near-field interference permeate the reconstructed far-field results; Abundant fake peaks emerge in the spatial spectrum and the direction of far-field targets is not accurately estimated.

**Figure 2:**
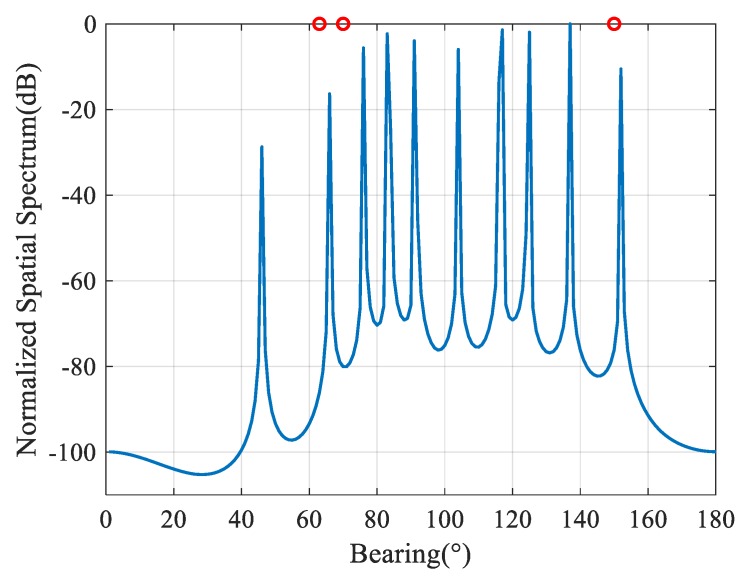
Spatial spectrum estimation of sparse Bayesian learning under two near-field interferences.

The spatial spectrum estimation result of the sparse Bayesian learning method of a ULA is simulated, which is shown in Figure 2. The red hollow circle represents the real far-field arrivals. There are two near-field interferences at (140.9°, 5.2λ) and (146.3°,2.7λ). The interference-to-noise ratio (INR) is set to 50 dB. The energy of near-field interferences permeates the far-field space, which creates several fake peaks in the far-field spatial spectrum based on sparse reconstruction.

### 4.2. Unified Sparse Representation Model for Far- and Near-field Sources

An over-complete model is established in this study to separate far-field targets from near-field interferences. the model incorporates the entirety of far-field and near-field by discretizing the spatial regions of far-field and near-field sources, respectively, giving the definition:

(15) $$\begin{array}{l} \Theta =\left\{\theta_{F_{1}},\ldots ,\theta_{Fi}\ldots ,\theta_{FN_{0}}\right\},1\leq i\leq N_{0},\\ \left(R,\Theta \right)=\left\{\left(r_{1},\theta_{1}\right),\ldots ,\left(r_{i},\theta_{j}\right),\ldots ,\left(r_{N_{1}},\theta_{N_{2}}\right)\right\},1\leq i\leq N_{1},1\leq j\leq N_{2},\\ A_{D}=\left[\begin{array}{cc} A_{F}(\Theta ) & A_{N}(R,\Theta ) \end{array}\right]\in C^{M\times N},N=N_{0}+N_{1}N_{2}. \end{array}$$

Here, the far-field space is quantized to $N_{0}$ at certain angle intervals, the whole far-field space is simulated with the angle set Θ, and the near-field space is discretized according to the 2D coordinate position. The whole near-field space is simulated with (R,Θ) and $A_{D}$ includes the steering vectors (far-field and near-field) of the whole space. Correspondingly, $A_{F}(\Theta )$ is the far-field over-complete dictionary, $A_{N}(R,\Theta )$ is the near-field over-complete dictionary and each column of $A_{D}$ is a basic function. Similarly, $s_{F}\left(t\right)$ and $s_{N}\left(t\right)$ in Equation (2) are sparsely extended in the spatial domain. 

Set $s_{D}\left(t\right)={\left[{\bar{s}}_{F}(t){\bar{s}}_{N}(t)\right]}^{T}\in C^{N\times 1}$, where the number of the non-zero elements of $s_{D}\left(t\right)$ is *K*, so that the array output can be expressed in an over-complete form:

(16) $$x\left(t\right)=A_{D}s_{D}\left(t\right)+n\left(t\right),\;t=1,\ldots ,T.$$

Equations (15) and (16) gives the uniform sparse representation of signals where both far-field targets and near-field interferences exist. The matrix form with the multiple measurement vector can be rewritten as:

(17) $$X=A_{D}S_{D}+N,$$

where $X=[x(t_{1}),\ldots ,x(t_{T})]$, $S_{D}=[s_{D}(t_{1}),\ldots ,s_{D}(t_{T})]$, and $N=[n(t_{1}),\ldots ,n(t_{T})]$. When targets and interferences are stationary relative to the receiving array during the observation, the number and position of non-zero elements in $s_{D}(t_{1}),\ldots ,s_{D}(t_{T})$ are constant. The following optimization problems can be solved to obtain the estimation results of the signal matrix $S_{D}$:

(18) $$ℒ\left(S_{D}|X\right)={\left\|X-A\left(\Theta \right)S_{D}\right\|}_{F}^{2}+\eta g\left(S_{D}\right).$$

In practical applications, although the location of near-field interference cannot be accurately obtained due to the complex distribution of noise sources, the pre-existing information regarding the region of near-field interferences can still be obtained. The positions of major noise sources such as propellers or motors are always relatively fixed. Therefore, to construct a complete dictionary for near-field interferences, the prior information of the interference locations should be properly accounted for to minimize the dictionary size and computational complexity of the reconstruction algorithm.

After correctly reconstructing the signal matrix $S_{D}$, the *K*_1_ far-field targets should be all composed of the basis of $A_{F}(\Theta )$ in the far-field dictionary and the energy of the other K_2_ near-field interferences should be fitted by the basis in the dictionary $A_{N}(R,\Theta )$. The far-field target arrival can be estimated free of the influence of the near-field interference based on the far-field spatial spectrum $P_{F}(\Theta )={\left[||S_{D}(1,:)|{|}_{2}^{2},\ldots ,||S_{D}(N_{0},:)|{|}_{2}^{2}\right]}^{T}$.

### 4.3. Sparse Bayesian Modeling 

Lp-norm penalization is a commonly used penalty function for the sparse constraint. Algorithms of this type typically require prior estimation of noise power, and/or the source number as input. In fact, algorithms using Lp-norm as sparse constraint penalty function are equivalent from the perspective of Bayesian estimation, assuming that the signal vectors obey the following prior-distribution:

(19) $$p\left(S_{D}\right)\propto \exp\left(\sum_{n=1}^{N}{\left\|S_{D}\left(n,:\right)\right\|}_{2}^{p}\right).$$

The probability density function in Equation (19) reaches its peak at 0. The function value is closer to 0 when the row vector in the signal matrix deviates farther from the zero vector. Assuming that the mean Gaussian noise is 0 and the variance is 1/η, the signal matrix ${\hat{S}}_{D}$ can be obtained via maximizing a posteriori (MAP):

(20) $$\begin{array}{l} {\hat{S}}_{D(MAP)}=\arg\underset{S_{D}}{\max}p(S_{D}|X)\\ \propto \arg\underset{S_{D}}{\min}=\eta {\left\|X-A_{D}\left(\Theta \right)S_{D}\right\|}_{F}^{2}+\sum_{n=1}^{N}{\left\|S_{D}\left(n,:\right)\right\|}_{2}^{p}. \end{array}$$

The convex relaxation algorithm based on the L1-norm is a special case of Equation (20) with *p* = 1. Equation (20) shows that the convex relaxation method essentially adds the same prior distribution assumption to the sources of different directions while the sparseness of the impinging signals requires a larger weight of $||S_{D}\left(n,:\right)|{|}_{2}^{p}$ corresponding to the real signal, and is smaller in other directions. When there is a large difference in the power of different spatial sources, adding the same weights to all signals will result in an additional suppression of strong signal components and crosstalk between different components, especially between far and near-field signal energies. These factors can make the reconstructed results inconsistent with the true distribution of signals.

The sparse Bayesian learning method [36] can be used to solve Equation (18) to mitigate the shortcomings of the Lp-norms method. Firstly, the observed noise is assumed to satisfy the zero-mean Gaussian distribution and variance of $\sigma_{n}^{2}$. The conditional probability of the array output data with respect to the signal matrix is:

(21) $$p\left(X|S_{D};\sigma_{n}^{2}\right)={\left(2\pi \sigma_{n}^{2}\right)}^{-MT/2}\exp\left(-\frac{1}{2\sigma_{n}^{2}}{\left\|X-A_{D}S_{D}\right\|}_{F}^{2}\right).$$

Corresponding to the signal matrix $S_{D}$, it is assumed that each row of elements obeys a Gaussian distribution with the mean value of 0 and the variance of $\gamma_{n}(n=1,\ldots ,N)$. The vector $\gamma ={\left[\gamma_{1},\ldots ,\gamma_{n},\ldots ,\gamma_{N}\right]}^{T}$ represents the power of the source at each discrete point in the whole space. Thus, each row of the matrix obeys a T-dimensional Gaussian distribution:

(22) $$p\left(S_{D}\left(n,:\right);\gamma_{n}\right)=N\left(0,\gamma_{n}I\right).$$

Unlike the assumption in Equation (19), each row of the signal matrix is assumed to conform to the Gaussian distribution of different parameters, thus avoiding the negative impact of L1-norm on all signals with constant weights. The noise power $\sigma_{n}^{2}$ and the signal prior-variance power γ are then assumed to satisfy the following gamma distribution:

(23) $$\begin{array}{l} p(\gamma )=\prod_{n=1}^{N}\mathrm{Gamma}\left(\gamma_{n}|a,b\right),\\ p(\sigma_{n}^{-2})=\mathrm{Gamma}\left(\sigma_{n}^{-2}|c,d\right). \end{array}$$

The probability density function of the Gamma distribution can be defined as:

(24) $$\mathrm{Gamma}\left(\gamma |a,b\right)=\frac{b^{a}}{\Gamma \left(a\right)}\gamma^{a-1}e^{-a\gamma },$$

where, $\Gamma \left(a\right)=\int_{0}^{\infty }t^{a-1}e^{-t}dt$ is the gamma function. According to Bayesian probability theory, the posterior probability density function of $S_{D}$ relative to X is:

(25) $$\begin{array}{ll} p\left(S_{D}|X;\sigma_{n}^{2}\right) & =\frac{p\left(X|S_{D};\sigma_{n}^{2}\right)p\left(S_{D};\gamma \right)}{\int p\left(X|S_{D};\sigma_{n}^{2}\right)p\left(S_{D};\gamma \right)dS_{D}}\\ & ={\left|\pi \mu_{S}\right|}^{-T}\exp\left\{-\mathrm{tr}\left[{\left(S_{D}-\mu_{S}\right)}^{H}\Sigma_{S}^{-1}\left(S_{D}-\mu_{S}\right)\right]\right\}, \end{array}$$

the mean and variance of which can be expressed as:

(26) $$\begin{array}{l} \mu_{S}=\Gamma A_{D}^{H}{\left(\sigma_{n}^{2}I+A_{D}\Gamma A_{D}^{H}\right)}^{-1}X,\\ \Sigma_{S}=\Gamma -\Gamma A_{D}^{H}{\left(\sigma_{n}^{2}I+A_{D}\Gamma A_{D}^{H}\right)}^{-1}A_{D}\Gamma , \end{array}$$

where Γ=diag(γ) is the diagonal matrix of vector γ. Given the γ and $\sigma_{n}^{2}$, by maximizing the posterior probability density function in Equation (25), the mean in Equation (26) can be exploited as the estimation value of the signal matrix. By removing the energy corresponding to the dictionary of near-field interference, the far-field spatial spectrum without near-field interferences can be obtained as follows:

(27) $$\mu_{F}\left(\Theta \right)={\left[{\left\|\mu_{S}\left(1,:\right)\right\|}_{2}^{2},\ldots ,{\left\|\mu_{S}\left(N_{0},:\right)\right\|}_{2}^{2}\right]}^{T}$$

To secure reasonable values of γ and $\sigma_{n}^{2}$, the likelihood function of γ and $\sigma_{n}^{2}$ can be expressed as:

(28) $$\begin{array}{ll} L\left(\gamma ,\sigma_{n}^{2}\right) & =-2\log p\left(X;\gamma ,\sigma_{n}^{2}\right)\\ & =-2\log\int p\left(X|S_{D};\sigma^{2}\right)p\left(S_{D};\gamma \right)dS_{D}\\ & =T\log\left|\Sigma_{X}\right|+X^{H}\Sigma_{X}^{-1}X \end{array}$$

where $\Sigma_{X}≜\sigma_{n}^{2}I+A_{D}\Gamma A_{D}^{H}$. The spatial spectrum is obtained by maximizing $L\left(\gamma ,\sigma_{n}^{2}\right)$ and substituting γ into Equations (26) and (27). The peak of the spatial spectrum is the estimation of the target arrivals.

### 4.4. Update of the Sparse Signal and Noise Power

When noise power $\sigma_{n}^{2}$ is known, the EM algorithm can be applied to optimize L(γ). Each iteration of the EM algorithm includes two steps: E-step and M-step. The posterior probability density expressed in Equation (26) is calculated in E-step. In M-step, the parameters γ can be updated as deterministic parameters by maximizing the likelihood function:

(29) $$\gamma_{i}^{\left(\mathrm{new}\right)}=\frac{1}{T}{\left\|\mu_{S}^{\left(\mathrm{old}\right)}\left(i,:\right)\right\|}_{2}^{2}+{\left(\Sigma_{S}^{\left(\mathrm{old}\right)}\right)}_{i,i},\forall i=1,\ldots ,N,$$

where $\gamma_{i}^{\left(\mathrm{new}\right)}$ represents the updated parameter values by the current iteration process, $\mu_{S}^{\left(\mathrm{old}\right)}$ and $\Sigma_{S}^{\left(\mathrm{old}\right)}$ are the mean and variance of the posterior probability of $S_{D}$ obtained by the previous iteration, respectively, and ${\left(\cdot \right)}_{i,i}$ is the ith element of the matrix. The fixed point iteration method can also be used to accelerate the convergence of the EM algorithm. The update strategy is:

(30) $$\gamma_{i}^{\left(\mathrm{new}\right)}=\frac{{\left\|\mu_{S}^{\left(\mathrm{old}\right)}\left(i,:\right)\right\|}_{2}^{2}/T}{1-{\left(\Sigma_{S}^{\left(\mathrm{old}\right)}\right)}_{i,i}/\gamma_{i}^{\left(\mathrm{old}\right)}}+\varsigma ,$$

where ς is set to a positive number close to 0 (in this paper, $\varsigma =10^{-8}$). 

When noise power $\sigma_{n}^{2}$ is unknown, the updated equation of $\sigma_{n}^{2}$ can also be obtained by $\partial L\left(\gamma ,\sigma_{n}^{2}\right)/\partial \sigma_{n}^{2}=0$.

(31) $${\left(\sigma_{n}^{2}\right)}^{\left(\mathrm{new}\right)}=\frac{{\left\|X-A_{D}S_{D}\right\|}_{F}^{2}/T}{M-N+\sum_{i=1}^{N}{\left(\Sigma_{S}\right)}_{i,i}/\gamma_{i}}.$$

However, in the actual application of the algorithm, the noise power as obtained by Equation (31) is often far smaller than the real noise power. This prevents the learning process of γ from effectively converging to the global optimal solution, and can even introduce irrelevant vectors to the reconstructed results. As mentioned above, false peaks degrade the estimation performance. 

In the EM algorithm architecture, an iterative approach to noise power estimation and sparse spectrum reconstruction can be deployed under a sparse Bayesian learning framework. The spatial spectrum estimation of each discrete point in space ($\gamma^{\left(\mathrm{new}\right)}$) can be obtained according to Equation (29) or (30) in each iteration. The number of spectral peaks is represented here as $\bar{K_{0}}$ and is arranged in descending order. The number of signals and interferences in space is considered to be less than the number of sensors. So $\bar{K}=\min\{\bar{K_{0}},M-1\}$ with the max-amplitude is saved as the estimated source number. The column index of the $\bar{K}$ peaks in the over-complete dictionary is Ω and the steering vector of the $\bar{K}$ sources with the largest power in this iteration is denoted by $A_{D}\left(\Omega \right)\in C^{M\times \bar{K}}$. 

Based on the coarse estimation results, the number of sources and the noise power can be estimated through information theory in each iteration. Assuming that the number of sources estimated in the current iteration is expressed as $\hat{K}$ and the unbiased estimation result of noise power can be expressed as follows:

(32) $$\hat{\sigma_{n}^{2}}=\frac{1}{T}{\left\|P_{A_{D}\left(\Omega \right)}^{⊥}\right\|}_{F}^{2}/\left(M-\hat{K}\right),$$

where $P_{A_{D}\left(\Omega \right)}^{⊥}=I-A_{D}\left(\Omega \right)A_{D}^{+}\left(\Omega \right)$ and $A_{D}\left(\Omega \right)$ is the source location estimation result. The estimated number of sources is as follows:

(33) $$\hat{K}=\arg\underset{k}{\min}\left[-\log p\left(X;\hat{\sigma^{2}}\right)+2kT+k+1\right]$$

where

(34) $$p\left(X;\hat{\sigma^{2}}\right)={\left|\pi \sigma^{2}\right|}^{-MT}\exp\left(-\sigma^{-2}{\left\|P_{A_{D}\left(\Omega \right)}^{⊥}\right\|}_{F}^{2}\right).$$

The noise power can be obtained by plugging the results of Equation (26) into Equation (25). If the number of sources is already known, the noise power can be estimated directly from Equation (25).

Based on the principle of the EM algorithm and the improved iterative process for noise power estimation presented above, we now summarize the proposed DOA estimation and parameter optimization algorithm like the following steps:

1. Determine the region of near-field interference according to the real array configuration.
2. According to Equation (15), sample the far-field and near-field spatial regions of interest separately at certain intervals to obtain $A_{F}(\Theta )$ and $A_{N}(R,\Theta )$. Construct the over-complete manifold dictionary of far-field and near-field hybrid models covering the whole space.
3. Initialize γ, and if the noise power is unknown, initialize $\sigma_{n}^{2}$.
4. Calculate $\mu_{s}$ and $\Sigma_{s}$ according to Equation (26).
5. Update parameter γ according to Equation (29) or (30).
6. If the number of sources is unknown, estimate the number of signals and interferences according to Equations (33) and (34), and update the noise power $\sigma_{n}^{2}$ by Equation (32) (or skip this step directly when the noise power is known).
7. Repeat Steps 4, 5 and 6 steps until γ and $\sigma_{n}^{2}$ converge to a fixed value.
8. Calculate the mean of the posterior probability $\mu_{S}$ of $S_{D}$ as the estimation of the signal amplitude matrix $S_{D}$. Finally, estimate the far-field signal direction after separation by Equation (27).

The over-complete dictionary $A_{D}$ consists of a far-field part and near-field part. After the sparse reconstruction of the signal using the proposed algorithm, the near-field point source interference steering vector usually has a stronger correlation with one or more vectors in the near-field dictionary. The energy of the interference source can then be fitted based on $A_{N}(R,\Theta )$. Similarly, the energy of the far-field signal is expressed by the basis in $A_{F}(\Theta )$, which separates the far-field signal from the near-field interference.

### 4.5. Complexity Analysis

For each iteration of the proposed algorithm, the computational complexity of the algorithm is dominated by evaluating the posterior probability density function in the E-step, computing the derivatives and updating the number of sources and noise power in the M-step. To obtain mean and variance of multivariate normal distribution expressed in Equation (26), it is required for computing the inversion of *N* × *N* matrix, which leads to the complexity of $O(N^{3})$. The calculation of $\mu_{S}$ presented in Equation (26) requires another $(M^{2}+M)N+MNT$ complex multiplications. The computing the derivatives of the objective function requires $O(MN^{2})$ and the calculation of $||P_{A_{D}\left(\Omega \right)}^{⊥}|{|}_{F}^{2}$ requires an inversion of M × M matrix, which leads to the complexity of $O(M^{3})$. Considering the spatial sparsity constraint and the limited number of snapshots, K<M≪N, T≪N, the computational complexity is $O(N^{3}+MN^{2})$. For L1-SVD, CBF, MVDR, and MUSIC, the computational cost is $O(N^{3})$, $O(M^{2})$, $O(M^{3})$, and $O(M^{3})$. 

It is worth mentioning that the superiority of the proposed algorithm comes at the cost of the increased computational complexity. It is a compromise between the performance of computational complexity when other state-of-art algorithms fail to provide satisfactory results.

## 5. Simulation, Experiment Results, and Discussion

Simulations and experiments were conducted to assess the performance of the proposed algorithm. In the simulations, CBF, MVDR, and MUSIC were compared with the proposed algorithm to observe the presence of near-field interference, the spatial spectrum of each algorithm, the probability of successful resolution and the RMS error of far-field direction estimation versus SNR and snapshot numbers.

Assume a ULA composed of 11 hydrophones with half-wavelength corresponding to the target signal sensor space. The reference sensor is located at the center of the array. Far-field signals and near-field interference exist simultaneously in the space, and the noise is additive Gaussian noise. The INR is defined as $10\log p_{k}^{2}/\sigma_{n}^{2}$, where $p_{k}^{2}$ is the received power of the kth near-field interference on the reference array.

### 5.1. Simulation Results of Spatial Spectrum Estimates

Three unrelated far-field narrowband signals impinge on the array with the same power from 63°, 70°, and 150°, respectively. There are two near-field interferences in the space and the positions relative to the central sensor are (−3.25λ,4λ) and (−1.5λ,2.25λ), the corresponding polar coordinates of which are (140.9°, 5.2λ) and (146.3°,2.7λ). Near-field interferences, far-field signals, and received noise are assumed to be unrelated to each other. The near-field region is sampled with (X,Y)={(x,y)|x=[−4λ:0.25λ:−1λ],y=[2λ:0.25λ:5λ]} and the sampling interval of the far-field is 1°, Θ=[1°:1°:180°]. The snapshot number is 200. 

**Figure 3:**
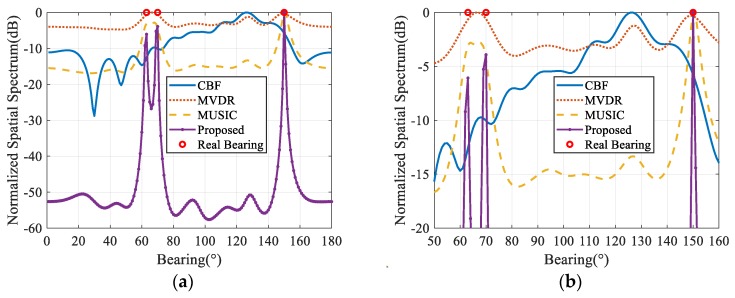

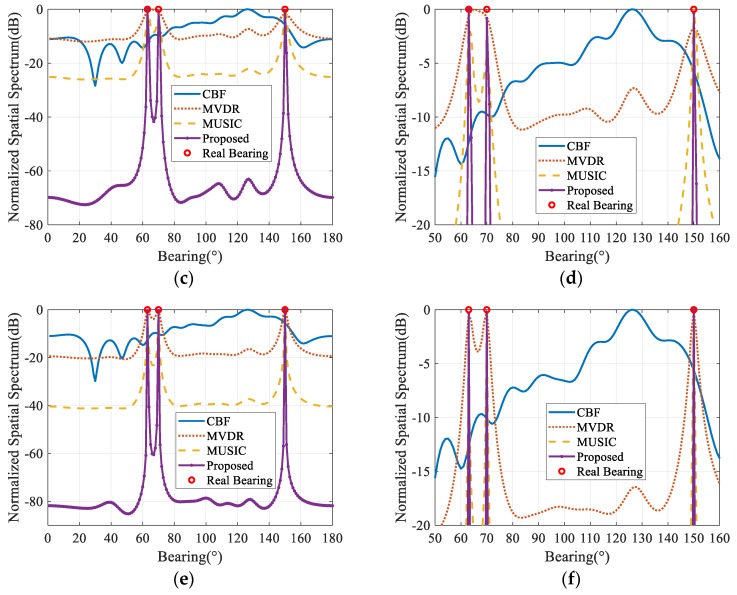
Spatial spectra under different signal-to-noise ratio (SNR) at interference-to-noise ratio (INR) of 50 dB. (**a**,**c**,**e**) Normalized spatial spectrum of conventional beamforming (CBF), Minimum Variance Distortionless Response (MVDR), Multiple Signal Classification (MUSIC), and the proposed method at SNR of −10 dB, 0 dB, and 10 dB, respectively; (**b**,**d**,**f**) partial enlargement at SNR of −10 dB, 0 dB, and 10 dB, respectively. Red hollow circles represent the real arrivals of far-field signals.

The number of near-field interferences plus far-field signals in the MUSIC algorithm is assumed to be prior known so that the signal plus interference subspace and noise subspace can be distinguished accurately. For other algorithms, there is no prior knowledge of noise power or source number. The spatial spectrum estimated results under simulation conditions of different algorithms are shown in Figure 3.

As shown in Figure 3, due to the presence of near-field strong interferences, the CBF method cannot accurately estimate the DOA of far-field sources even in the case of high SNR due to the leakage of interference energy from sidelobes. The signals from 63° and 70° are completely concealed by the energy leaked from the near-field interferences’ sidelobes, which results in the basic failure of the algorithm in the presence of near-field intensity interference.

The MVDR algorithm can restrain the interference from unknown directions adaptively, including near-field interference when designing beams, thus yielding better spatial spectrum results than the CBF algorithm. However, under low SNR level (<0 dB), the adaptive beamforming algorithm does not have the ability to distinguish the two targets from 63° and 70°. Further, the position of near-field interference is close to the far-field target from 150°, which weakens the interference suppression ability of beams near 150°. The spectrum under low SNR does not form the sharp peaks and deviates from the real DOA of the signals. However, these problems grow less intense as SNR increases. 

The MUSIC algorithm utilizes the orthogonality of the signal subspace and noise subspace to enhance the resolution to adjacent targets compared with MVDR. Its spatial spectrum estimation performance is also better than that of MVDR. However, under the low SNR level, the target from 150° also shows peak-broadening characteristics; further, the MUSIC algorithm is very sensitive to the prior-number of interferences and signals.

**Figure 4:**
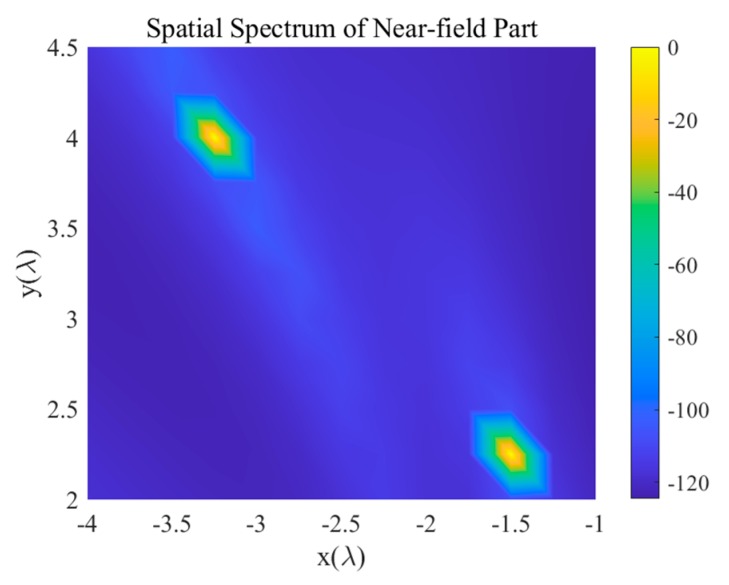
The spatial spectrum of the near-field part under SNR of 10 dB and INR of 50 dB.

Under the simulated SNR and INR levels, the proposed algorithm gives the most stable far-field spatial spectrum estimation and has the sharpest peaks among the algorithms simulated in this study. In addition, only the proposed algorithm can distinguish the two targets from 63° and 70° and correctly estimate all three target arrivals under the SNR of −10 dB. Unlike the MUSIC method, the proposed algorithm does not need the number of signals and interferences as its prior-input. Instead of suppressing the near-field interferences, the proposed algorithm effectively separates the far-field targets signals from the near-field interferences thanks to the high resolution of the sparse Bayesian learning framework. The estimated spatial near-field spectrum is shown in Figure 4, where the interference energy is well-constrained in the near-field steering vector dictionary. Therefore, the mutual crosstalk of far and near-field is avoided.

### 5.2. Convergence Analysis

**Figure 5:**
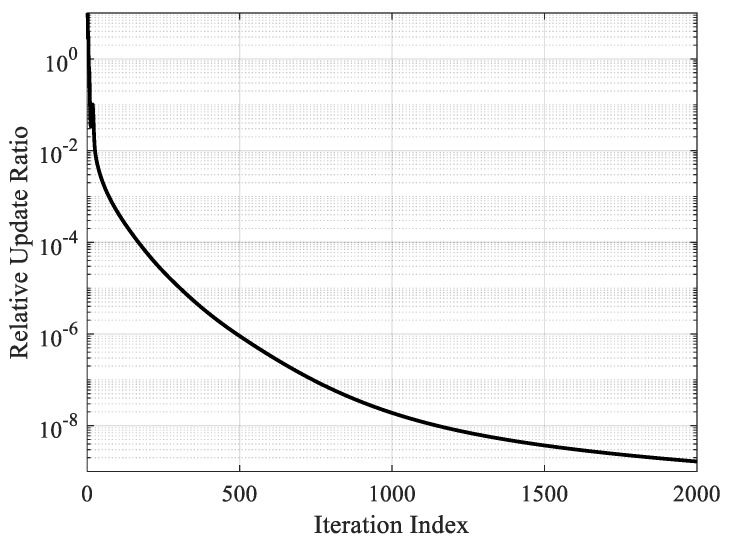
The convergence of the spatial spectrum iterative estimation process.

The proposed spatial spectrum sparse reconstruction algorithm iteratively optimizes the signal power spectrum and noise power under the framework of the EM algorithm. The convergence of the iterative optimization strategy and EM algorithm has been verified in many pieces of literature. In order to verify the convergence of the proposed DOA estimation algorithm, the relative update ratio of the signal power spectrum defined by $||\gamma^{\left(i+1\right)}-\gamma^{\left(i\right)}|{|}_{2}/||\gamma^{\left(i\right)}|{|}_{2}$ during 2000 iterations is calculated and shown in Figure 5.

As shown in Figure 5, due to the randomness of the initialization process, the first few iterations were not stable and the estimated spatial spectrum appears jitter. After multiple iterations, the estimated results quickly stabilize and show a tendency of monotonic convergence.

### 5.3. Statistical Performance Analysis of Simulation Results

The root mean square error (RMSE) of DOA estimation versus SNR and the number of snapshots were next investigated over 500 independent Monte Carlo simulations. To adjust the resolution of various algorithms with adjacent angles, two far-field signals were selected with DOAs of 90° and 130°; this prevented the large RMSE due to the algorithm’s poor resolution. One near-field source interference, in this case, was located at (−3.25λ,4λ), as mentioned above, the INR was 50 dB. The snapshot number was 200 and the SNR level was adjusted from −10 dB to 20 dB. The RMSE of azimuth estimation was calculated as follows:

(35) $${\mathrm{RMSE}}_{\theta }=\sqrt{\frac{1}{K\times \Omega }\sum_{i=1}^{\Omega }\sum_{k=1}^{K}{\left({\hat{\theta }}_{ki}-\theta_{k}\right)}^{2}},$$

where Ω is the number of Monte Carlo simulations, K is the number of sources, $\theta_{k}$ is the kth real direction of the signal, and ${\hat{\theta }}_{ki}$ is the estimated direction of the kth source in the ith Monte Carlo simulation. There were severe energy leaks of near-field interferences through the sidelobes of CBF, causing the algorithm to fail. The CBF was not subjected to any subsequent statistical analysis for this reason. 

**Figure 6:**
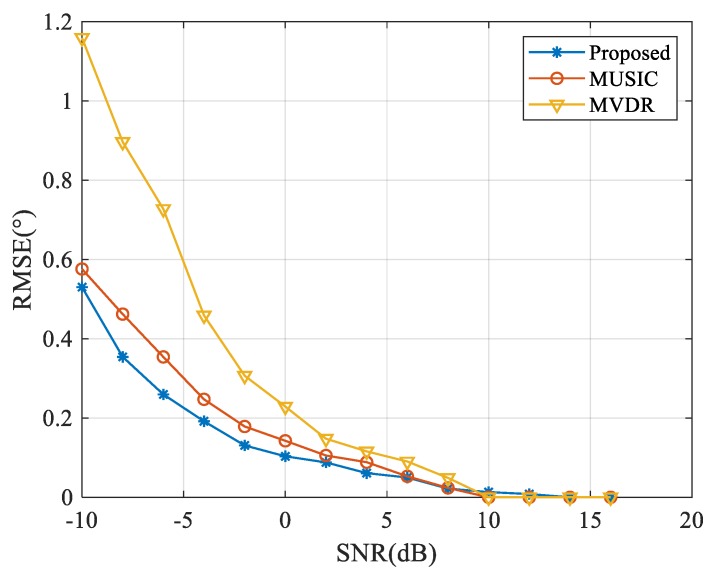
Results of root mean square error (RMSE) versus SNR under near-field INR of 50 dB.

The RMSEs of DOA estimation versus SNR with MVDR, MUSIC and the proposed algorithm are shown in Figure 6. The RMSE appears to decrease gradually as the SNR of the far-field signals increases. When the SNR of the far-field signals is low, the presence of the interference seriously affects the accuracy of DOA estimation. The RMSE of the proposed algorithm has an obvious advantage with lower SNR. When the SNR is sufficiently high, each algorithm presents nearly the same accuracy. As the SNR level continues to rise, the estimation accuracy of each algorithm tends toward stability due to the quantization error of the angle searching-set.

**Figure 7:**
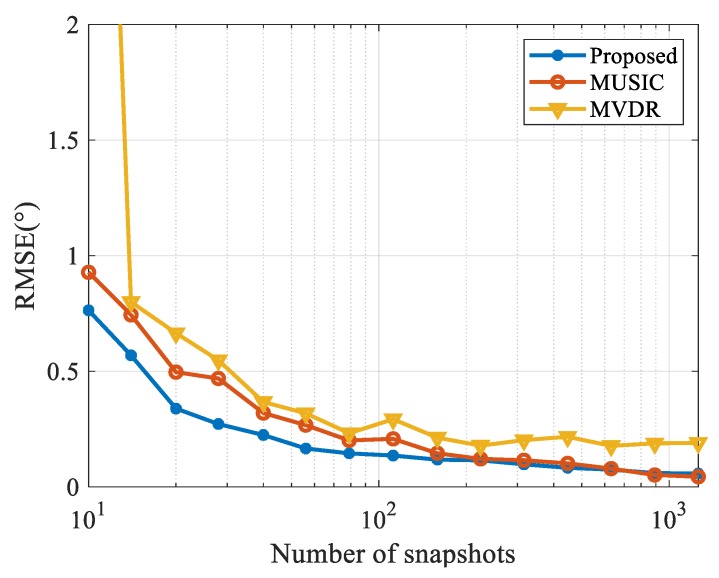
Results of RMSE versus snapshots under near-field INR of 50 dB.

The RMSE of each algorithm versus the number of snapshots (10 to 1260) under SNR of 0 dB is shown in Figure 7. Accurately estimating the covariance matrix of receiving data is one of the important prerequisites for the MUSIC or MVDR algorithms to function properly. A shortage of snapshots increases the error of the covariance matrix, thus driving down the accuracy of their estimations. The proposed method directly processes the array receiving data, so it performs well even when there is a scarcity of snapshots. When the number of snapshots is sufficient, the proposed algorithm performs as well as MUSIC. 

As shown in Figure 6 and Figure 7, the proposed algorithm can enhance the DOA estimation performance under low SNRs even with insufficient samples in the presence of near-field strong interference.

**Figure 8:**
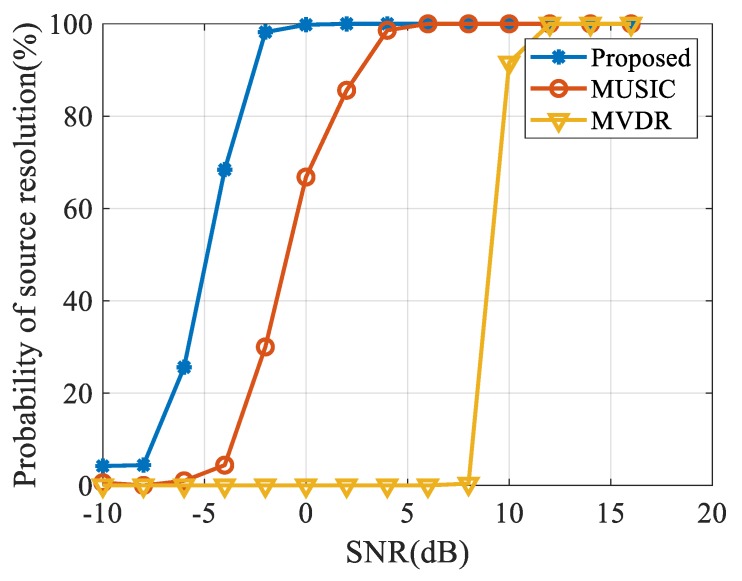
Results of the probability of source resolution versus SNR.

We also investigated the resolution of each algorithm to adjacent targets. The angles of the two far-field signals, in this case, were 90° and 94°, respectively. The snapshot number was 200 and the SNR varied from −10 dB to 20 dB. We ran 500 Monte Carlo simulations under each SNR level and determined the resolution of dual targets as:

(36) $$\frac{P({\hat{\theta }}_{1})+P({\hat{\theta }}_{2})}{2}>P\left(\frac{{\hat{\theta }}_{1}+{\hat{\theta }}_{2}}{2}\right),$$

where $P({\hat{\theta }}_{1})$ and $P({\hat{\theta }}_{2})$ are the peaks of the DOA estimation of the two targets respectively. The probability of source resolution is the percentage between iterations that satisfy Equation (29) and entire simulations. As shown in Figure 8, when the two targets have an angle interval of 4° and SNR is 0 dB, the proposed method has a 100% probability of source resolution while MUSIC and MVDR do not achieve any satisfactory resolution. 

**Figure 9:**
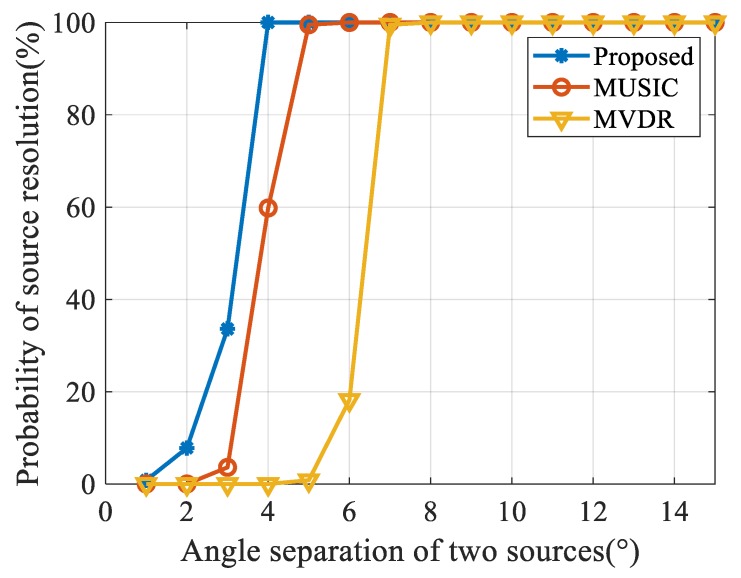
Results of the probability of source resolution versus angle separation.

We next fixed the location of the targets from 90° and varied the angle of the other source from 91° to 105°. The probability of source resolution for each algorithm versus the angle interval of the two targets with 0 dB SNR and 200 snapshots is shown in Figure 9. Under the specified conditions, each algorithm can distinguish dual targets with an angle interval of 7°, however, the angle interval threshold of the proposed algorithm is only 4°, which is lower than other algorithms. 

The simulations conducted in this study show that properly accounting for the sparsity constraint during spatial spectrum estimation can significantly enhance algorithm performance. The proposed algorithm thus has a lower SNR threshold and angle interval threshold in resolving the adjacent targets in space compared to other state-of-art algorithms. 

### 5.4. Experimental Data

**Figure 10:**
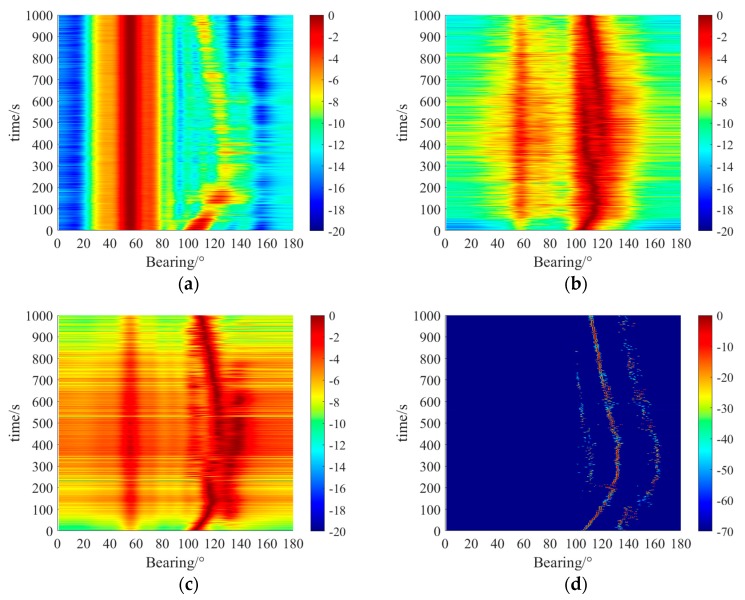
Bearing time recordings of (**a**) CBF, (**b**) MVDR, (**c**) MUSIC, (**d**) proposed algorithm.

We also conducted an in-situ experiment to verify the proposed algorithm. Experimental data was collected with 10 acoustic hydrophones uniformly spaced at 4 m. The array was placed at 10 m under the sea surface. The observed space [0°, 180°] was uniformly discretized at 1° interval where 0° is the end-fire direction. There was interference at the distance of 40 m and the direction of around 60°. A few weak targets moved around 100° to 160° throughout the experiment. The near-field interference region was discretized with (X,Y)={(x,y)|x=[16m:1m:32m],y=[24m:1m:40m]}. The received data was sampled at 2048 Hz. The duration of every processing segment was 2 s and the total analysis time was about 16 min. The CBF, MVDR, MUSIC, and proposed algorithms were applied to estimate the directions of the weak targets with an analyzed frequency of 180 Hz. The near-field interference was fixed relative to the receiving array throughout the 1000 s data-processing time. The results are shown in Figure 10. 

As shown in Figure 10a, the CBF algorithm is severely affected by near-field interference due to its lack of interference suppression measures. CBF was generally unable to detect the targets around 100° to 160° and the widths of the peaks are much wider than the other three algorithms, which indicates that its resolution is the poorest among the four DOA algorithms we tested. As shown in Figure 10b,c, the MVDR and MUSIC algorithm both distinguished the weak targets due to the suppression of near-field interference. The MUSIC algorithm has as mentioned above, requires prior knowledge of the number of targets.

As shown in Figure 10d, the strongest interference around 60° was completely eliminated by the separation of far-field and near-field signals by the proposed algorithm. The spatial spectrum of the residual far-field weak signals was reconstructed effectively. The trajectory is the clearest with the sharpest spectral peak. To this effect, the proposed DOA estimator outperformed the other algorithms we tested in the strong near-field interference environment.

### 5.5. Prospects for Future Work

The algorithm is proposed to solve the problem of high-resolution DOA estimation of far-field targets in presence of near-field interferences. In practical sonar system applications, how to achieve fast implementation of the sparse spectrum reconstruction is a practical problem. Strong demand for wideband signal processing and vector hydrophone array processing under random interferences still exists. How to use prior signal information such as signal structure is an open challenge.

## 6. Conclusions

Local interferences like towing vessels or propeller vibration noise in the array mounted on an underwater vehicle can be expressed as intensive near-field sources, which poses a severe challenge to the accurate DOA estimation of far-field targets. Far-field target detection and DOA estimation problems in the near-field interference environment were investigated in this study. The far-field target location estimation problem under near-field interference was transformed into a problem of separating far-field and near-field sources; a sparse Bayesian learning algorithm was proposed in this paper based on this idea. 

The spherical wave propagation model was first applied to simulate the near-field source, which prevents any model error otherwise caused by Fresnel approximation. A unified sparse representation model of far-field and near-field mixed sources was constructed considering the correlation difference between the planar waveguide vector and the spherical waveguide vector. The sparseness of the Bayesian model was then exploited to fit and constrain the array data while the high resolution of the sparse reconstruction algorithm was used to constrain the energy of near-field interference in the preset near-field steering vector over-complete dictionary. This, as demonstrated by simulation and experimental results, ensures the accurate detection of far-field targets. The number of sources, the power of noise, and the sparse reconstruction of signals are solved by joint iteration in the proposed sparse optimization process. The prior information such as the number of sources and power of noise is thus unnecessary and parameter adjustment is not needed.

Simulation and experimental results validated the effectiveness of the proposed algorithm in the presence of intensive near-field interference. The performance of the proposed algorithm in an experimental case study that has been carried out is better than similar state-of-the-art algorithms in the perspectives of SNR level, the number of samples and angle resolution.
